# Vitamin D Status Is Negatively Correlated with Insulin Resistance in Chinese Type 2 Diabetes

**DOI:** 10.1155/2016/1794894

**Published:** 2016-06-20

**Authors:** Jie Zhang, Jianhong Ye, Gang Guo, Zhenhao Lan, Xing Li, Zhiming Pan, Xianming Rao, Zongji Zheng, Fangtao Luo, Luping Lin, Zhihua Lin, Yaoming Xue

**Affiliations:** ^1^Department of Endocrinology & Metabolism, Nanfang Hospital, Southern Medical University, Guangzhou, Guangdong 510515, China; ^2^Xiamen Second Hospital affiliated Xiamen Medical College, Xiamen 361021, China; ^3^Sanming City Hospital of Integrated Traditional Chinese and Western Medicine, Sanming 365000, China; ^4^Department of Endocrinology, The Second Hospital of Shanxi Medical University, Taiyuan 030001, China

## Abstract

*Objectives*. Vitamin D deficiency plays a role in insulin resistance and the pathogenesis of type 2 diabetes mellitus. Little information is available about the association between vitamin D status and insulin resistance in the Chinese population. Currently, vitamin D status is evaluated by the concentrations of serum 25-hydroxyvitamin D [25(OH)D]. This study explores the relationship between insulin resistance and serum 25-hydroxyvitamin D concentrations in Chinese patients with type 2 diabetes mellitus.* Subjects and Methods*. This study included 117 patients with type 2 diabetes. The following variables were measured: 25-hydroxyvitamin D [25(OH)D], glycosylated hemoglobin A1c (HbA1c), fasting blood glucose (FBS), fasting blood insulin (FINS), fasting blood C-peptide, serum creatinine (SCr), glomerular filtration rate (eGFR), body mass index (BMI), and homeostatic model estimates of insulin resistance (HOMA-IR).* Results*. The cases were divided into three groups: Group 1 (G1) with 25(OH)D ≤ 20 ng/mL [≤50 nmol/L], Group 2 (G2) with 25(OH)D values from 20 ng/mL [50 nmol/L] to 30 ng/mL [75 nmol/L], and Group 3 (G3) with 25(OH)D ≥ 30 ng/mL [≥75 nmol/L], with 52.6%, 26.3%, and 21.1% of subjects in Groups 1–3, respectively. There was a negative correlation between 25(OH)D and HOMA-IR (*β* = −0.314, *p* = 0.001) adjusted by age, BMI, and eGFR.* Conclusion*. Better vitamin D status may be protective of glucose homeostasis since 25(OH)D was negatively associated with insulin resistance in Chinese patients with type 2 diabetes.

## 1. Introduction

Diabetes is becoming a chronic, global epidemic with accelerated morbidity, increased and earlier mortality, and increased healthcare costs. Total global diabetes prevalence is ~9.7%, with 92.4 million adults with diabetes [1]. China is one of the countries with the heaviest diabetes burden, and the prevention and control of this disease are difficult tasks for Chinese society. Type 2 diabetes is a progressive and chronic disease characterized by both *β*-cell dysfunction and increased insulin resistance, defined as the inadequate response of skeletal muscle, liver, and adipose tissue to endogenous insulin secretions, and few drugs ameliorate increases in insulin resistance.

Recently, considerable interest has been generated on the extraskeletal and nonclassical effects of vitamin D [2, 3] based on the presence of vitamin D receptors (VDRs) and the activating hydroxylase in target tissues other than bone, gut, and kidney. Animal and human studies suggest that vitamin D may play a role in modulation of the risk of diabetes [4]. Subjects at risk of diabetes had lower 25(OH)D concentrations than those without risk [5, 6]. Vitamin D deficiency was related to the presence and prospective development of type 2 diabetes mellitus [4, 7–11], and compared to subjects without diabetes, type 2 diabetes patients had lower serum 25(OH)D [12]. The relationship between vitamin D status and the risk of type 2 diabetes or insulin resistance was reported in several studies. Vitamin D deficiency can play a role in insulin resistance and the pathogenesis of type 2 diabetes, through effects on both *β*-cell function and insulin sensitivity [13, 14]. Several roles of vitamin D deficiency are reported affecting insulin resistance through various mechanisms including increasing related proinflammatory cytokines and acute phase reactants, as found in vitamin D deficiency, mediating low-grade inflammation [15–17]. However, data on the inverse association between 25(OH)D concentrations and the development of insulin resistance (IR) is conflicting. In Europeans with metabolic syndrome, vitamin D status may not correlate with insulin activity or secretion [18], and relationships between vitamin D status and IR differ among different racial groups. Moreover, little research is available on the association between insulin resistance and vitamin D status in the Chinese population. As a result, this relationship needs to be specifically explored in different populations, including the Chinese. The aim of this research, therefore, was to clarify the relationship between vitamin D status and insulin resistance in Chinese people with T2DM. Currently, vitamin D status is evaluated by the concentration of total 25-hydroxyvitamin D [25(OH)D] [19]. According to Endocrine Society Clinical Practice Guidelines on vitamin D deficiency, serum circulating 25-hydroxyvitamin D [25(OH)D] concentrations were measured to evaluate vitamin D status [20]. We hypothesise that 25(OH)D concentrations negatively correlate with insulin resistance status based on Scragg et al. [21] and Chonchol and Scragg [22] researches.

## 2. Subjects and Methods

### 2.1. Materials and Methods

Based on Chiu et al.'s research [14], we identified 117 patients with type 2 diabetes who were treated in the outpatient Department of Endocrinology and Metabolism of Xiamen Second Hospital from January 2014 to March 2014. Three of the included subjects refused 25(OH)D concentration and intact parathyroid hormone test. The following criteria were used to include patients: (i) age ranging from 20 to 70 years, (ii) history less than 10 years, (iii) serum parathyroid hormone concentration ranging from 15.0 to 65.0 pg/mL, (iv) a serum calcium concentration less than 2.45 mmol/L, (v) normal routine blood tests of liver function, serum creatinine, and normal electrolytes, and not being treated with insulin or with thiazolidinedione (TZD), vitamin D, or drugs modulating vitamin D efficacy. Major reasons for excluding individuals included (i) absence of type 2 diabetes or presence of diabetic ketoacidosis, ketonuria, or diabetic hyperosmolar syndrome, (ii) serum phosphorus > 1.60 mmol/L, (iii) acute infection, (iv) tumors, and (v) pregnant or nursing women.

### 2.2. Anthropometric and Biochemical Analysis

Data were gathered on age, sex, and family history of type 2 diabetes. Participants' weights and heights were recorded, and body mass index (BMI) was calculated. The body weights and heights were measured while the subjects wore light clothing without shoes. The BMI was calculated as the weight in kilograms divided by the height in meters squared. Systolic blood pressure (SBP) and diastolic blood pressure (DBP) were measured while sitting, after a 5 minute rest, and again after a 10 minute interval, and the mean values were recorded. Hypertension was also diagnosed by a history of hypotensive medication. Blood samples were drawn between 08:00 am and 09:00 am for laboratory analysis of biochemical variables [25-hydroxyvitamin D (25(OH)D), glycated hemoglobin A1c (HbA1c), fasting blood glucose (FBS), fasting insulin (FINS), fasting serum C-peptide, total cholesterol (TC), triglyceride (TG), high-density lipoprotein (HDL-C), low-density lipoprotein (LDL-C), intact parathyroid hormone (iPTH), urea nitrogen (BUN), serum creatinine (SCr), serum calcium (Ca), and serum phosphorus (P)]. Intact parathyroid hormone (iPTH) (ntact parathyroid hormone kits, Beckman Coulter; Beckman DXI800 access immunoassay system), FINS [insulin kits, Roche Diagnostics (Shanghai); Roche Cobas 6000 analyzer], C-peptide (C-peptide kits, Roche Diagnostics; Roche Cobas 6000 analyzer), and serum 25(OH)D (25-hydroxyvitamin D kits, Roche Diagnostics; Roche Cobas 6000 analyzer) concentrations were determined by the electrochemical luminescence method. Fasting blood glucose (blood glucose kits, Beijing Leadman; Beckman DXI800 access immunoassay system) was measured by the oxygen electrode method. Serum creatinine (creatinine kits, Beijing Leadman Biochemistry; Siemens ADVIA 2400 automatic biochemical analyzer), TC (total cholesterol kits, Beijing Leadman Biochemistry; Siemens ADVIA 2400 automatic biochemical analyzer), TG (triglyceride kits, Beijing Leadman Biochemistry; Siemens ADVIA 2400 automatic biochemical analyzer), LDL (low-density lipoprotein kits, Sekisui Medical Japan; Siemens ADVIA 2400 automatic biochemical analyzer), and HDL (high-density lipoprotein kits, Randox UK; Siemens ADVIA 2400 automatic biochemical analyzer) were measured by the enzymatic method. HbA1c (D-10 glycosylated hemoglobin kits, Bio-Rad; Bio-Rad Dias TAT glycosylated hemoglobin analyzer) was measured by High Performance Liquid Chromatography (HPLC). BUN (urea nitrogen kits, Ningbo Medical System; Siemens ADVIA 2400 automatic biochemical analyzer) was measured by the urease method. Serum calcium (calcium kits, Ningbo Medical System; Siemens ADVIA 2400 automatic biochemical analyzer) and serum phosphorus (phosphorus kits, Siemens China; Siemens ADVIA 2400 automatic biochemical analyzer) were measured by the ion selective electrode method. Vitamin D deficiency was defined as 25(OH)D of ≤20 ng/mL [≤50 nmol/L], vitamin D insufficiency was defined as a 25(OH)D between 20 ng/mL [50 nmol/L] and 30 ng/mL [75 nmol/L]. The normal serum 25(OH)D concentration was defined as ≥30 ng/mL [≥75 nmol/L], according to Endocrine Society Clinical Practice Guideline of vitamin D deficiency [20]. HOMA-IR was calculated from fasting insulin and fasting glucose. HOMA-IR had the formula fasting glucose (mmol/L) × fasting insulin (pmol/L)/22.5 [23]. eGFR was calculated by the MDRD GFR equation [24]. As this was a retrospective study, and the data were anonymously analysed, informed consent was unnecessary.

### 2.3. Statistics

SPSS 19.0 software was used for statistical analysis. Continuous variables were expressed as the mean ± SD (mean standard deviation). Least-Significant Difference test (LSD-*t*) was used to analyse the comparison of 25(OH)D and *β*-cell function indices (FINS, HbA1c) for G1, G2, and G3 subjects. Independent-sample *t* test was used to compare 25(OH)D, HOMA-IR, and glucose metabolism indices (FBS, FINS, and HbA1c) in male and female subjects. Multiple linear regression analysis was used to examine the association between serum 25(OH)D concentration and insulin resistance, HOMA-IR, analysed as dependent variable with the other significantly associated variables [25(OH)D, eGFR, BMI, and age] as independent variables, and a *p* value < 0.05 was considered significant.

## 3. Results

### 3.1. Baseline Characteristics

A total of 117 patients were enrolled in this study; three of them refused 25(OH)D concentration and iPTH test.  Table 1 shows the characteristics of the study subjects and their metabolic index levels. The population had an even sex distribution (53.85% male) and a normal BMI (mean BMI = 24.90 ± 3.24 kg/m^2^) and was middle aged (mean age = 50.38 ± 13.47 years). The average serum 25(OH)D value was 21.40 ± 10.68 ng/mL (53.1 ± 26.35 nmol/L), below the sufficiency cutoff value of 30 ng/mL (75 nmol/L). The prevalence of vitamin D deficiency [25(OH)D < 50 nmol/L] was 52.6% of the total study subjects.

### 3.2. Comparison of G1, G2, and G3 of HOMA-IR and Glucose Metabolism Indices (FINS, HbA1c)

The subjects were divided into three groups according to serum 25(OH)D value: (G1) vitamin D deficiency [25(OH)D ≤ 20 ng/mL (50 nmol/L)], (G2) vitamin D insufficiency [25(OH)D value between 20 ng/mL (50 nmol/L) and 30 ng/mL (75 nmol/L)], and (G3) normal vitamin D status [25(OH)D ≥ 30 ng/mL (75 nmol/L)]. The results of the analyses comparing data for G1, G2, and G3 are given for the mean ± SD in Table 2; the mean HOMA-IR was significantly different between all the three groups, as shown in Table 2, and both HOMA-IR and fasting insulin values were lower with higher mean 25(OH)D concentrations.

### 3.3. Comparison of Male and Female of 25(OH)D, Glucose Metabolism Indices (FBS, FINS, and HbA1c), and HOMA-IR

The values for serum 25(OH)D and for insulin resistance and glucose indices (fasting blood glucose, fasting insulin levels, and HbA1c) by gender are presented in Table 3. 25(OH)D values, HOMA-IR, fasting blood glucose, and fasting insulin levels were not significantly different in male and female subjects, but the mean HbA1c was significantly higher in men than that in women, as shown.

### 3.4. Relationship between Serum 25(OH)D Levels and HOMA-IR

**Figure 1:**
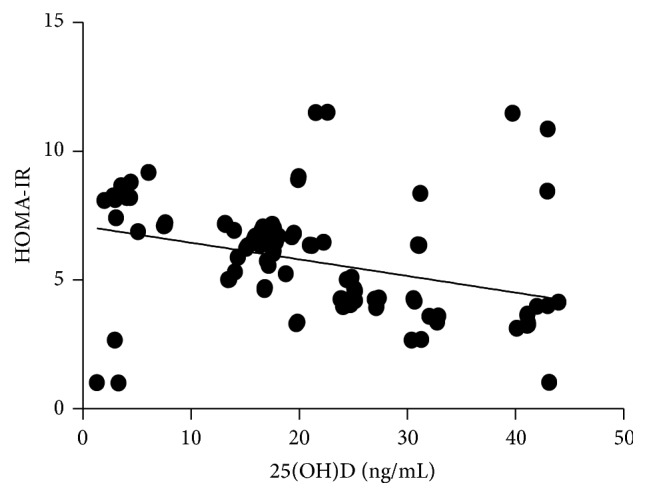
Inverse association between 25(OH)D and HOMA-IR (Pearson correlation = −0.327, *p* = 0.000).

In the multiple linear regression analysis (shown in Table 4), vitamin D status was a predictor of HOMA-IR, as the dependent variable, but not eGFR, BMI or age, as independent variables. The Pearson correlation coefficient showed that 25(OH)D had a negative correlation with HOMA-IR (regression coefficient *r* = −0.327, *p* = 0.000; see Figure 1).

## 4. Discussion

The prevalence of type 2 diabetes mellitus (T2DM) has increased significantly and globally over the last few decades. Type 2 diabetes results from insulin resistance linked to dyslipidemia, hyperglycemia, obesity, and hypertension. Few drugs are clinically effective in ameliorating insulin resistance. Vitamin D is a well-known vitamin responsible for bone and calcium metabolism [7]. Its deficiency results in rickets in children and osteomalacia in adults. Cutaneous synthesis and dietary ingestion are the main sources of vitamin D [25, 26]. The proportion of serum vitamin D intake from the diet is small [27, 28], and it is mainly provided by sun exposure during outdoor activities. In recent years, the extraskeletal effects of vitamin D have attracted much interest [2, 3]. Lower serum 25(OH)D concentrations are associated with higher risks for the development of type 2 diabetes mellitus [8]. In addition, more type 2 diabetes patients than control subjects had vitamin D insufficiency and deficiency [29, 30]. 25(OH)D values were lower in type 2 diabetes mellitus patients than in control group, and poor vitamin D status [25(OH)D values] was associated with less good glycemic control in type 2 diabetes mellitus patients [5, 25]. The Australian diabetes study showed that 25(OH)D data were inversely associated with type 2 diabetes risk in their population [31]. Many in vitro studies suggest repletion of vitamin D insufficiency may improve insulin secretion and sensitivity [32, 33]. However, reports on associations between insulin secretion or insulin resistance and 25(OH)D have been inconsistent among different racial groups. Vitamin D deficiency has also been suggested to be related to insulin resistance and to the risks of type 2 diabetes mellitus, as well as those of metabolic syndrome, but 25(OH)D values may not correlate with insulin activity or secretion [4, 7–11]. Moreover, few studies are available on the association between insulin resistance and vitamin D status in the Chinese population. Most research has investigated vitamin D metabolism in Chinese women with gestational diabetes or with metabolic syndrome [34–36], but one study from Cai et al. found no correlation between 25(OH)D values and insulin resistance [37], though exclusion criteria, such as treatment with insulin and thiazolidinedione administration, were omitted in that cross-sectional study which may have affected the results.

Our cross-sectional study suggests that, amongst our subjects with type 2 diabetes, the proportions with vitamin D deficiency group (G1), vitamin D insufficiency (G2), and normal vitamin D status (G3) were 52.6%, 26.3%, and 21.1%, respectively. All the subjects lived in southern China (northern latitude 24.27°, east longitude 118.06°) and, despite sufficient sunlight, more than half of the subjects were vitamin D deficient, and only 21.1% were “replete.” The prevalence of vitamin D deficiency in patients with type 2 diabetes will probably be higher in north China as this area gets less sunshine than southern China. Both HOMA-IR and fasting insulin values decreased with higher 25(OH)D concentrations, in all the three groups. Insulin resistance and the associated biomarkers were negatively associated with 25(OH)D status in our representative population of Chinese patients with type 2 diabetes, other than eGFR, BMI, and age. Similar data has been found in other racial groups. An inverse association of insulin resistance with 25(OH)D concentration has been found for 25(OH)D values between 16 and 36 ng/mL [38]. Another study suggested that 25(OH)D possibly modulated glycemic responses, both in impaired glycemia and in healthy subjects [39]. Other studies have indicated a role for vitamin D supplementation in modulation of insulin resistance and improvement of its resultant complications. In Von Hurst et al., insulin resistance was reduced but only if serum 25(OH)D on supplementation reached >80 nmol/L (>32 ng/mL) [40]; and although few data demonstrated that vitamin D insufficiency was associated with insulin resistance [21, 41–43], it was suggested that adequate vitamin D status may be helpful for prevention of increases in insulin resistance and of subsequent T2DM.

The role of vitamin D deficiency in insulin resistance is thought to have several potential mechanisms, including increasing the formation of proinflammatory cytokines and acute phase reactants, likely to increase low-grade inflammation, as well as the well-known promotion of insulin secretion from beta-cells [14, 15, 17]. The efficacy of vitamin D in stimulating insulin release can be affected by vitamin D axis gene polymorphisms, such as those for the activating enzyme (vitamin D 1*α*-hydroxylase; CYP27B1) and the transport protein (vitamin D binding protein), as well as for vitamin D receptors (VDR) [44–49]. Moreover, hypocalcemia worsens effects of vitamin D deficiency, including decreasing further the glucose-stimulated release of insulin from *β*-cells [50]. Lower serum 25(OH)D concentrations also increase serum parathyroid hormone (PTH), itself leading to decreased glucose uptake by liver, muscle, and adipose cells [51]. In another study, variants of the vitamin D receptors (VDRs) in pancreatic *β*-cells, skeletal muscle, and adipose tissue were associated with variation in the effects of vitamin D on glucose metabolism. Insulin sensitivity was affected by the presence of 1*α*-hydroxylase in *β*-cells and the presence of vitamin D response element in the human insulin receptor gene promoter region. The transcription of peroxisome proliferator activator receptor (PPAR) *γ* and of the human insulin receptor gene was directly activated by calcitriol; in vitro, vitamin D acceleration of glucose transport was mediated by insulin and vitamin D activated insulin receptor gene transcription [52–55]. Certain vitamin D binding proteins and allelic variations in the vitamin D receptor gene might also affect insulin secretion and glucose tolerance, which, in turn, might contribute to the genetic risk of type 2 diabetes [56]. Data on the MRC-Ely prospective study demonstrated that the baseline 25(OH)D concentration was inversely related to insulin sensitivity and glycemia [57, 58]. Further research is needed to explore whether vitamin D deficiency results in insulin resistance and whether supplementing with vitamin D or its active metabolite may prevent, or ameliorate, insulin resistance in healthy people or in Chinese people with type 2 diabetes.

## 5. Conclusion

Vitamin D status possibly plays a role in maintaining glucose homeostasis. There is a negative correlation between circulating levels of 25(OH)D and insulin resistance in Chinese people with type 2 diabetes.

## Figures and Tables

**Table 1:** Characteristics of the study population.

Variables		*n*
Age (years)^a^	50.38 ± 13.47	117
Men^c^	53.85	63
Body mass index (kg/m^2^)^a^	24.90 ± 3.24	117
Systolic blood pressure (mmHg)^a^	125.76 ± 14.79	117
Diastolic blood pressure (mmHg)^a^	80.85 ± 9.30	117
Fasting plasma glucose (mmol/L)^a^	8.23 ± 3.28	117
HOMA-IR^a^	5.79 ± 2.14	117
C-peptide (*μ*IU/mL)^a^	2.09 ± 1.04	117
25(OH)D (ng/mL)^a^	21.40 ± 10.68 (53.1 ± 26.35 nmol/L)	114
Vitamin D deficiency^c^	52.6	60
Vitamin D insufficiency^c^	26.3	30
Normal 25(OH)D value^c^	21.1	24
HbA1c (%)^a^	9.51 ± 2.81	117
iPTH (pg/mL)^a^	43.07 ± 14.21	114
TC (mmol/L)^a^	5.14 ± 1.12	117
LDL (mmol/L)^a^	3.06 ± 1.01	117
Ca (mmol/L)^a^	2.33 ± 0.14	117
P (mmol/L)^a^	1.36 ± 0.15	117
eGFR (mL/min·1.73 m^2^)^b^	111.70 [93.85–131.35]	117
History (year)^b^	3.00 [0.00–8.00]	117
FINS^b^	117.10 [72.30–156.52]	117
TG^b^	1.65 [1.06–2.82]	117
HDL^b^	1.23 [1.11–1.46]	117
BUN^b^	4.81 [4.13–5.69]	117
SCr^b^	56.50 [48.05–70.45]	117

Notes: ^a^mean ± SD, ^b^median [interquartile range], and ^c^percentage.

HOMA-IR: homeostatic model estimates of insulin resistance, 25(OH)D: 25-hydroxyvitamin D, TC: total cholesterol, LDL: low-density lipoprotein, Ca: serum calcium, P: serum phosphorus, eGFR: glomerular filtration rate, FINS: fasting blood insulin, TG: triglyceride, HDL: high-density lipoprotein, BUN: blood urea nitrogen, SCr: serum creatinine, and HbA1c: glycated hemoglobin A1c, iPTH: intact parathyroid hormone.

**Table 2:** Comparison of G1, G2, and G3 of HOMA-IR and glucose metabolism indices (FINS, HbA1c).

	G1	G2	G3	*p*_G1G2_ value	*p*_G2G3_ value	*p*_G1G3_ value
HOMA-IR	6.44 ± 1.70^*∗*※^	5.00 ± 1.94^*∗*^	4.80 ± 2.57^※^	0.006	0.204	0.000
FINS (*μ*U/mL)	141.20 ± 72.59^※^	128.14 ± 56.79^#^	93.23 ± 65.82^#※^	0.590	0.010	0.001
HbA1c (%)	10.08 ± 2.78^*∗*^	7.87 ± 2.34^*∗*#^	9.99 ± 2.89^#^	0.000	0.003	0.955

Notes: ^*∗*^comparison of G1 and G2 is significant; ^#^comparison of G2 and G3 is significant; ^※^comparison of G1 and G3 is significant; HOMA-IR: homeostatic model estimates of insulin resistance; FINS: fasting blood insulin; HbA1c: glycated hemoglobin A1c; *p*_G1G2_: *p* value of G1 and G2 comparison; *p*_G2G3_: *p* value of G2 and G3 comparison; *p*_G1G3_: *p* value of G1 and G3 comparison.

**Table 3:** Comparison of male and female of 25(OH)D, glucose metabolism indices (FBS, FINS, and HbA1c), and HOMA-IR.

	Male	Female	*p* value
25(OH)D (ng/mL)	20.46 ± 11.76	22.55 ± 9.16	0.301
HOMA-IR	5.78 ± 2.16	5.81 ± 2.12	0.953
FBS (mmol/L)	8.03 ± 3.76	8.47 ± 2.64	0.452
FINS (*μ*U/mL)	134.72 ± 75.47	118.78 ± 59.14	0.203
HbA1c (%)	10.09 ± 3.09	8.83 ± 2.28	0.013

Notes: 25(OH)D: 25-hydroxyvitamin D, HOMA-IR: homeostatic model estimates of insulin resistance, FBS: fasting blood glucose, FINS: fasting blood insulin, and HbA1c: glycated hemoglobin A1c.

**Table 4:** Multiple linear regression analysis between HOMA-IR and 25(OH)D, eGFR, BMI, and age.

	HOMA-IR	
	*β*	*p* value
25(OH)D	−0.314	0.001
Age	0.077	0.446
eGFR	−0.059	0.546
BMI	0.191	0.043

Note: HOMA-IR used as dependent variable and 25(OH)D, eGFR, BMI, and age used as independent variables.
